# Supplementary material for: Reverse engineering morphogenesis through Bayesian optimization of physics-based models
Source: NPJ Syst Biol Appl. 2024 May 7;10:49. doi: 10.1038/s41540-024-00375-z (PMC11076624; doi:10.1038/s41540-024-00375-z)
Supplement: Supplementary file 1 — Supplementary Information [file 41540_2024_375_MOESM1_ESM.pdf]

Reverse engineering morphogenesis through Bayesian optimization of physics-based models

Nilay Kumar[1*], Mayesha Sahir Mim[1,2**], Alexander Dowling[1***], Jeremiah Zartman[1,2****]

[1]Department of Chemical and Biomolecular Engineering, University of Notre Dame, Notre Dame IN, 46556
[2]Bioengineering Graduate Program, University of Notre Dame, Notre Dame IN, 46556
(* - nkumar4@nd.edu, ** - mmim@nd.edu, *** - adowling@nd.edu, **** - jzartman@nd.edu)

Supplementary Tables

Supplementary Table 1: Table corresponding to Figure 4C

$log(k_A^{col})$	$log(k_B^{col})$	$log(k_L^{col})$	$log(L0_A^{col})$	$log(L0_B^{col})$	$log(L0_L^{col})$	$log(K_{ECM})$	F_B	*Group*
-0.1457	-6.4092	1.7781	-2.7738	-1.5063	-0.0522	-11.2278	0.09940	Control-1
-0.1457	-6.4092	1.7781	-2.7738	-1.5063	-0.0522	-11.2278	0.0994	Control-1
-0.551	-3.1890	-1.7789	-2.9772	-1.3214	-0.0258	-13.1908	0.1042	Control-1
0.1594	-5.3218	-0.0223	-2.8371	-1.4229	-0.1745	-15.2949	0.1083	Control-1
0.0510	-4.1404	-3.3223	-2.9066	-1.8135	-0.3121	-15.2864	0.1096	Control-1
-0.3332	-5.2156	-0.9684	-2.9125	-1.4194	0.00271	-7.5177	0.1153	Control-1
-0.2216	-4.7614	-5.2241	-2.8923	-1.2269	-0.5623	-16.6767	0.1165	Control-1
0.2265	-5.4243	-0.3725	-2.8076	-2.2940	-0.0523	-14.9203	0.1166	Control-1
-7.4342	1.5032	-1.6709	-1.2590	-1.7627	0.7887	-13.9026	0.1113	Control-2
-7.4342	1.5032	-1.6709	-1.2590	-1.7627	0.7887	-13.9026	0.1113	Control-2
-8.27186	2.1807	-0.5177	-1.3336	-1.7698	0.6753	-18.1196	0.1122	Control-2
-11.2967	-8.6174	-3.9609	-2.2909	-2.4713	0.8820	-17.9534	0.2348	Clgns
-11.2967	-8.6174	-3.9609	-2.2909	-2.4713	0.8820	-17.9534	0.2348	Clgns
-8.87599	-6.75723	-5.89843	-2.6936	-1.6007	0.6700	-7.1689	0.2386	Clgns
-11.2266	-6.86542	-7.56488	-2.2389	-2.1785	0.9723	-11.9379	0.2390	Clgns
-11.2266	-6.86542	-7.56488	-2.2389	-2.1785	0.9723	-11.9379	0.2390	Clgns

Supplementary Table 2: Table corresponding to Figure 5C

$log(k_A^{col})$	$log(k_B^{col})$	$log(k_L^{col})$	$log(L0_A^{col})$	$log(L0_B^{col})$	$log(L0_L^{col})$	$log(K_{ECM})$	F_B	***Group***
-8.3067	-3.3898	-7.1575	-1.707	-1.6217	0.8230	-8.0974	0.1045	Case I
-8.3097	-11.2236	-11.1142	-1.7834	-2.3492	0.0376	-12.4807	0.1099	Case I
-9.2125	-0.4135	0.3028	-1.8481	-1.6338	0.7404	-4.9901	0.1019	Case I
-11.3945	-0.4277	-10.324	-1.3742	-1.3303	1.2532	-4.7872	0.0824	Case II
-10.3464	2.0910	-4.4691	-1.5400	-1.6170	1.1392	-5.1180	0.0818	Case II
-8.8677	2.1366	-2.0050	-1.8368	-1.4873	1.1661	-6.0692	0.0891	Case II
-10.5006	1.8216	-2.2324	-1.9311	-1.4193	1.1403	-5.9649	0.0821	Case II
-9.7547	-0.4106	-0.4306	-1.5697	-1.7461	0.9965	-5.192	0.0693	Case II
-3.6974	1.5870	-7.4071	-1.8436	-2.2116	1.1328	-15.5845	0.1209	Case III
-6.5249	1.1620	-7.3866	-1.5486	-2.3996	1.2288	-18.2644	0.1140	Case III
-7.7476	0.1057	-6.9335	-2.8963	-2.8410	1.2445	-13.6869	0.1162	Case III
-9.3780	1.5621	-3.8466	-1.2561	-2.9289	0.9043	-17.9181	0.1357	Case IV
-8.9513	1.7643	-0.9020	-1.8360	-2.9622	0.9749	-13.3347	0.1197	Case IV
-8.9194	1.9544	-3.7264	-1.5244	-2.9255	0.9263	-13.3634	0.1360	Case IV
-9.9168	2.0507	-1.7202	-2.2472	-2.8870	0.9852	-18.2693	0.1197	Case IV

Supplementary Table 3: Glossary of abbreviations and acronyms

**Computational Terms**	
GPR	Gaussian Process Regression
MLE	Maximum Likelihood Estimation
MAP	Maximum A Posteriori
MCMC	Markov Chain Monte Carlo
SMC-ABC	Sequential Monte Carlo- Approximate Bayesian Computations
SCE	Subcellular Element
BO	Bayesian Optimization
EFD	Elliptic Fourier Descriptors
DTW	Dynamic Time Warping
LHS	Latin Hypercube Sampling
SE	Surface Evolver
EI	Expected Improvement
pdf	Probability Distribution Function
cdf	Cumulative Distribution Function
**Experimental Terms**	
AP	Anterior-Posterior
DV	Dorsal-Ventral
AEL	After Egg Laying
ECM	Extracellular Matrix
MMP1	Matrix Metallopeptidase 1
pMyoII	Phosphorylated Non-Muscle Myosin II
ROCK	Rho-Kinase

Supplementary Figures

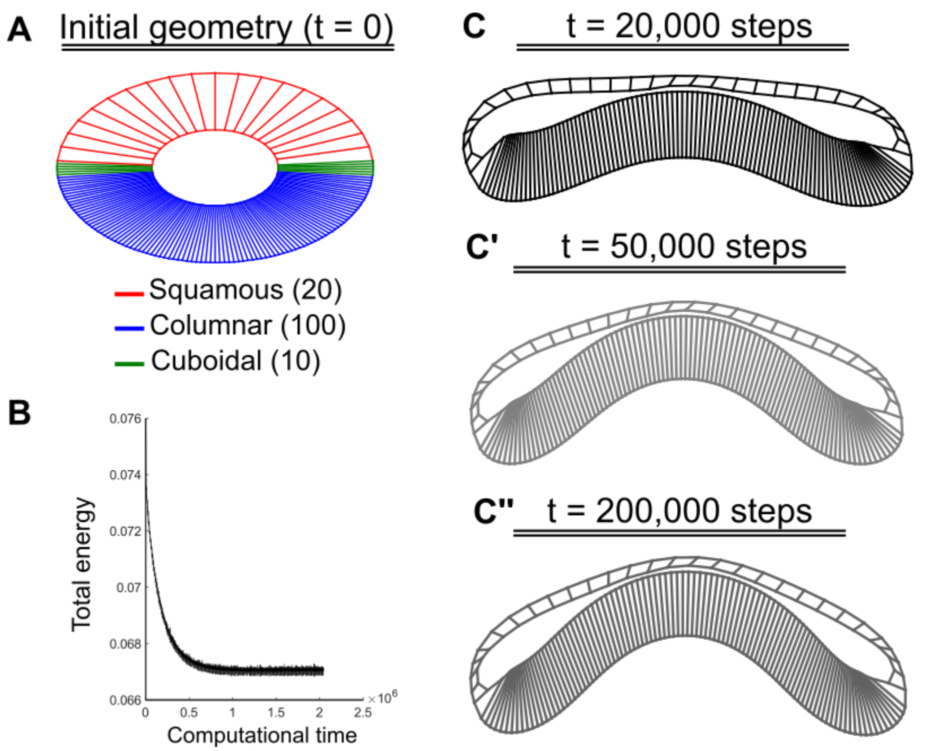

Supplementary Figure 1: Convergence of Surface Evolver simulations. (A) The initial geometry used for modeling the AP cross-section of the wing disc resembles a donut. Parameters are specified differently for the different cell types named squamous, columnar, and cuboidal. **(B)** Plot showing variation in total energy with computational time. The energy reduces initially until it converges. **(C-C'')** Shape changes in the simulation cross-section with computational time. With energy, the shape also converges leading to a local minimum configuration.

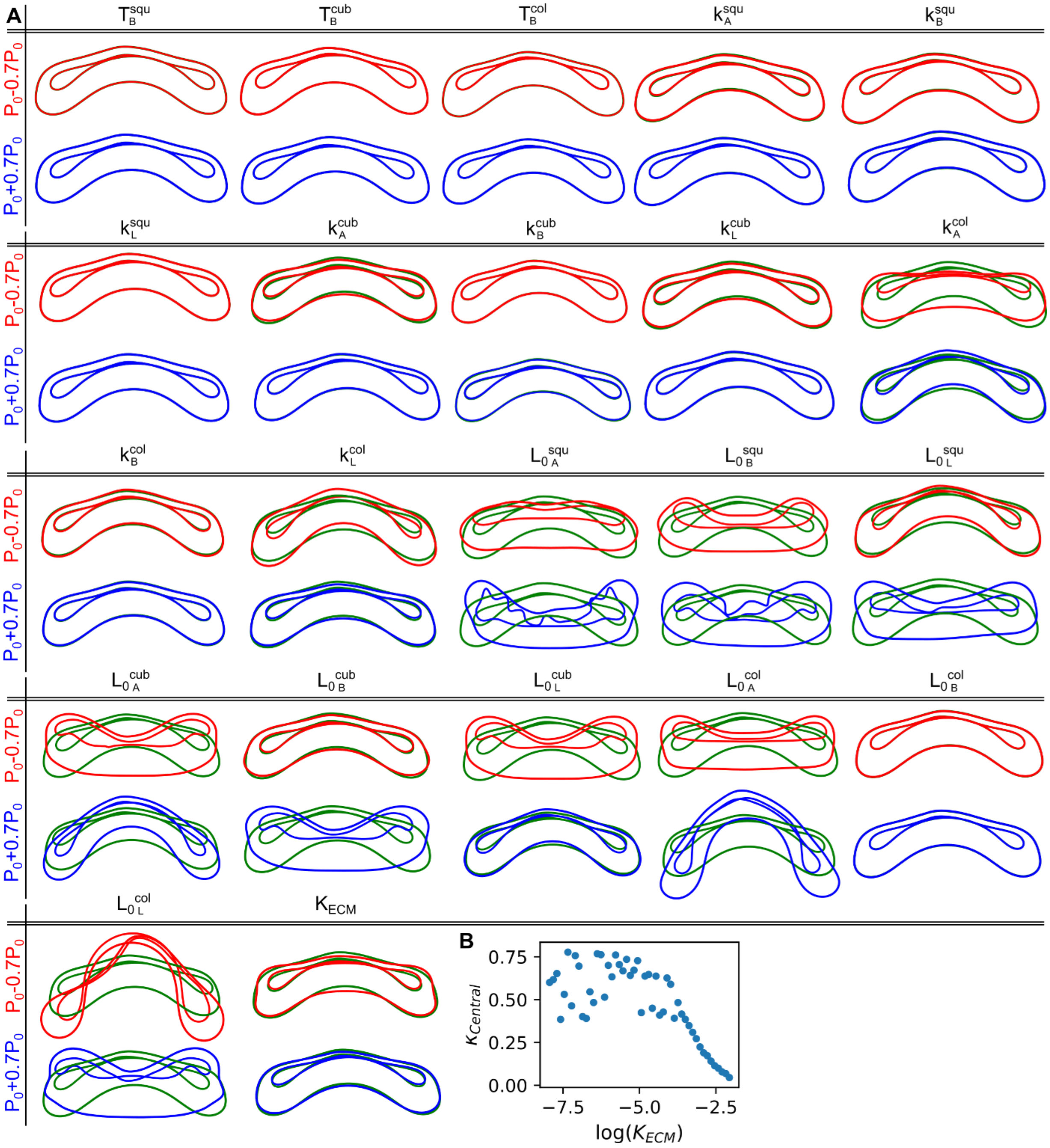

Supplementary Figure 2: Shape changes in wing imaginal disc as a result of perturbing individual parameters of the Surface Evolver model one at a time. Individual model parameters within the model were increased and decreased by 70%, and Surface Evolver was run to measure the changes in tissue shape as a result of perturbation. The green contours within the plots indicate the base case. Blue and Red contours refer to the cases where the model parameters were increased and decreased respectively. (B) 50 points were sampled by varying model parameters k_B^{col} and K_{ECM}. Scatter plot showing variation of basal central curvature ($\kappa_{Central}$) with log(K_{ECM}) (Refer Figure 2B for more details).

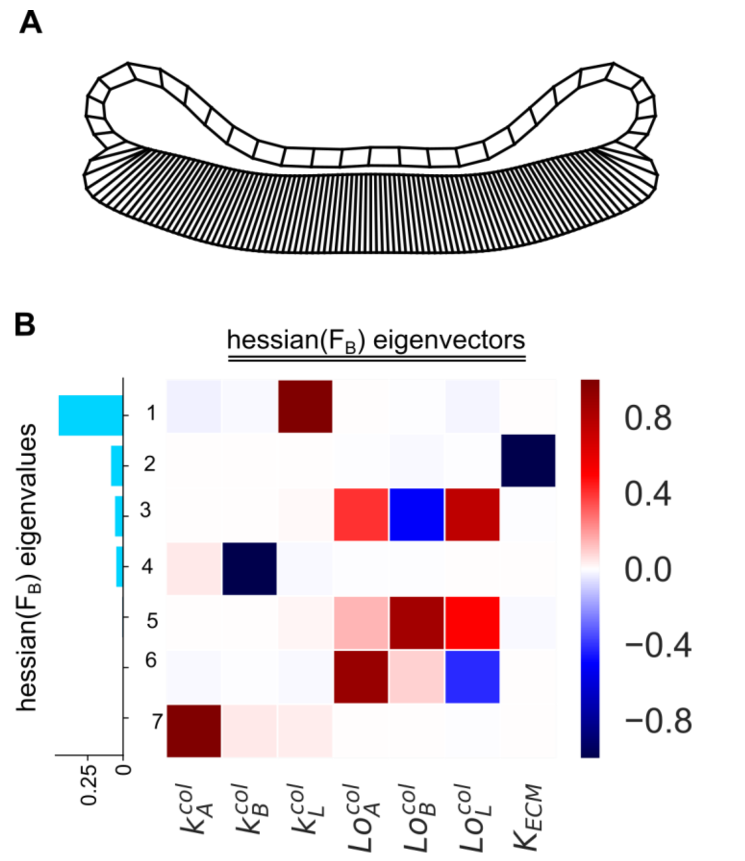

Supplementary Figure 3: (A) Surface Evolver was used to simulate the minimum energy configuration for the average value of the parameters sampled (Table S1, Case: Clgns) using BO corresponding to the input wing disc cross-section shown in Figure 4A’ (B) Eigendecomposition analysis of the local Hessian of F_B. Each row of the heatmap corresponds to the eigenvector of the Hessian matrix. The rows are arranged in decreasing order of the corresponding eigenvalues represented by a bar plot on the left-hand side of the heatmap.

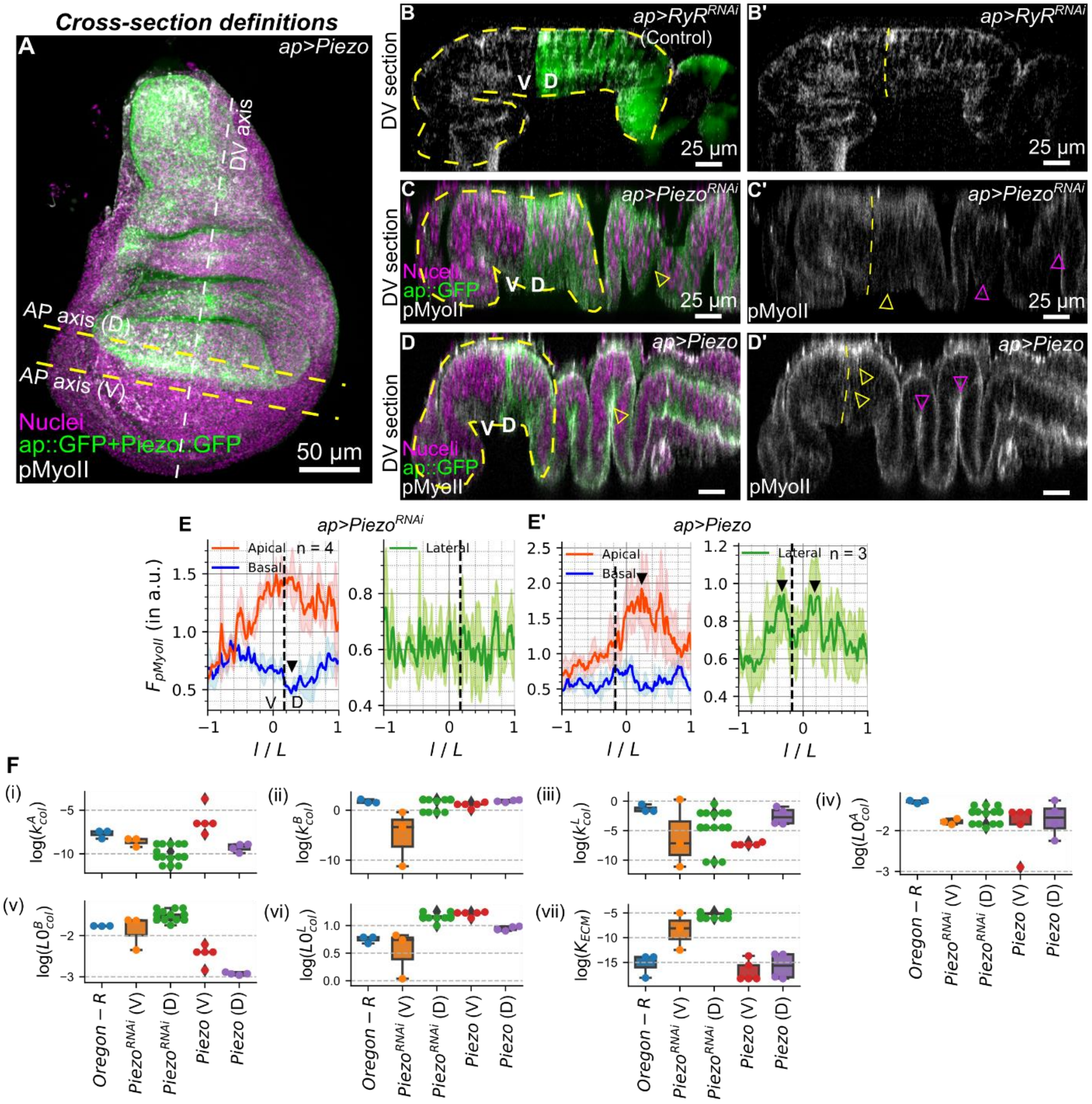

Supplementary Figure 4: Piezo regulates pMyoII expression during wing imaginal disc development. An ap-Gal4 driver was used to downregulate and overexpress Piezo in the dorsal compartment of the wing disc using *UAS-Piezo^{RNAi}* and *UAS-Piezo:GFP* (BDRC# 58772) transgenic fly lines respectively. **(A)** A maximum intensity z-plane projection of the disc expressing *ap>Piezo*. Different lines within the pouch indicate the approximate location of the cross-section analyzed in Figure 5. **(B-B')** DV cross-section of the disc expressing *ap>RyR^{RNAi}* (BDRC# 31540). *ap>RyR^{RNAi}* has been used as a control for generating mutants as Ryanodine receptor (RyR) is not known to be expressed in the wing imaginal disc tissue[1] **(C-C')** DV cross-section of the disc expressing *ap>Piezo^{RNAi}* (VDRC# 105132). **(D-D')** DV cross-section of the disc expressing *ap>Piezo:GFP* (BDRC# 58772). Fluorescent labels have been indicated within the plot. **(E,E')** Quantifying normalized fluorescent intensity of pMyoII along the apical-basal and lateral surfaces of the pouch. The x-axis of the pouch represents the normalized distance from the pouch center where the fluorescent intensity was calculated. Normalization of fluorescent intensity has been carried out by dividing the apical-basal signals with the max reported intensity value in the basal surface. A solid colored line represents the mean, and the shaded region

represents the standard deviation in prediction from multiple samples. **(F)** (i-vii) Box plot showing the variation of parameters (x-axis) for the best shapes predicted by the BO framework for AP cross sections presented in Figure 5. Parameters of the best shape predictions for the AP section of the Oregon-R disc shown in Figure 4A have been used as a control for comparison. The ventral compartments are also interpreted as an internal control, given that the perturbation does not explicitly change Piezo expression levels in that compartment. It should be noted that the variation of parameters is lower for Oregon-R. Additionally, since tissue mechanics is interconnected, comparing the ventral compartment with Oregon-R may not be informative, as perturbations in one compartment can influence the biomechanics of the other.

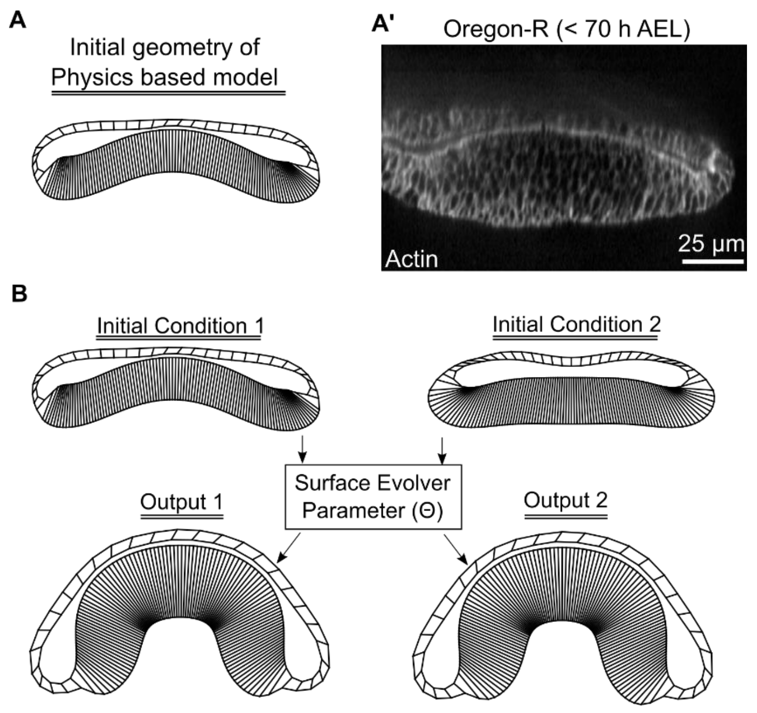

Supplementary Figure 5: Output is robust to changes in the initial shape. (A) Initial geometry of the wing disc used in the physics-based model. **(A')** AP cross-section of the early stage wing imaginal disc. Genotype and staging time have been indicated on the top panel. **(B)** Two separate simulations were run by varying the initial geometry (top panel). The plot's bottom panel shows output geometries due to energy minimization. The parameters used for running the two simulations were unchanged.

Supplementary References

1. Consortium, T. modENCODE *et al.* Identification of Functional Elements and Regulatory Circuits by Drosophila modENCODE. *Science* **330**, 1787–1797 (2010).
